# Chicken interferon alpha pretreatment reduces virus replication of pandemic H1N1 and H5N9 avian influenza viruses in lung cell cultures from different avian species

**DOI:** 10.1186/1743-422X-8-447

**Published:** 2011-09-22

**Authors:** Haijun Jiang, Hanchun Yang, Darrell R Kapczynski

**Affiliations:** 1 Key Laboratory of Zoonosis of Ministry of Agriculture, College of Veterinary Medicine and State Key Laboratory of Agrobiotechnology, China Agricultural University, Beijing, People's Republic of China; 2 Exotic and Emerging Avian Disease Unit, Southeast Poultry Research Laboratory, Agricultural Research Service, USDA, 934 College Station Road, Athens, GA, 30605, USA

**Keywords:** avian influenza, interferon, chicken, duck, turkey

## Abstract

**Background:**

Type I interferons, including interferon alpha (IFN-α), represent one of the first lines of innate immune defense against influenza virus infection. Following natural infection of chickens with avian influenza virus (AIV), transcription of IFN-α is quickly up regulated along with multiple other immune-related genes. Chicken IFN-α up regulates a number of important anti-viral response genes and has been demonstrated to be an important cytokine to establish anti-viral immunity. However, the mechanisms by which interferon inhibit virus replication in avian species remains unknown as does the biological activity of chicken interferon in other avian species.

**Methods:**

In these studies, we assessed the protective potential of exogenous chicken IFN-α applied to chicken, duck, and turkey primary lung cell cultures prior to infection with the pandemic H1N1 virus (A/turkey/Virginia/SEP-4/2009) and an established avian H5N9 virus (A/turkey/Wisconsin/1968). Growth kinetics and induction of select immune response genes, including IFN-α and myxovirus-resistance gene I (Mx), as well as proinflammatory cytokines (IL-1β and IL-6), were measured in response to chicken IFN-α and viral infection over time.

**Results:**

Results demonstrate that pretreatment with chicken IFN-α before AIV infection significantly reduced virus replication in both chicken-and turkey-origin lung cells and to a lesser degree the duck-origin cells. Virus growth was reduced by approximately 200-fold in chicken and turkey cells and 30-fold in duck cells after 48 hours of incubation. Interferon treatment also significantly decreased the interferon and proinflammatory response during viral infection. In general, infection with the H1N1 virus resulted in an attenuated interferon and proinflammatory response in these cell lines, compared to the H5N9 virus.

**Conclusions:**

Taken together, these studies show that chicken IFN-α reduces virus replication, lower host innate immune response following infection, and is biologically active in other avian species.

## Background

Avian influenza (AI) is a viral disease of poultry that can occur in many different bird species, with wild aquatic birds, including ducks, considered the natural reservoir for the AI viruses in the environment [1]. Both high and low pathogenic avian influenza viruses are continually being isolated from wild and domestic species of birds, causing concern of outbreaks in the poultry industry. In addition, recent outbreaks of human infections caused by influenza viruses containing genes of avian lineage, including H1N1, H5N1, H7N2, H7N3, H7N7, and H9N2, demonstrates that AI viruses can be transmitted directly to humans from domestic poultry [2]. Thus, domestic poultry can act as intermediate hosts for the transmission of influenza viruses from wild aquatic birds to humans due to the inherent closeness of rearing.

Interferons (IFNs) are a group of polypeptides that are secreted from most all eukaryotic cells in response to external signals. They are classified into three groups, designated type I, type II and type III. Type I IFN (α and β), are expressed rapidly after viral infection, and represent a first line of defense initiated by the innate immune response. Chicken type I IFN (ChIFN) was the first IFN to be discovered over 50 years ago and was described as a virus-induced factor able to interfere with influenza virus replication in chorioallantoic membranes of chicken embryos [3]. IFNs generally have been considered to be host species specific, yet it is known that several IFN proteins show various degrees of cross-species activity. Turkey IFN-α shares 91% and 82% identity with chicken IFN-α at the nucleotide (nt) and amino acid (aa) sequence levels, respectively. Duck IFN (DuIFN) is 73% identical to the ChIFN at the nt level but only 50% identical at the aa level [4]. Bertram et al. reported functional homology in supernatants of PHA-stimulated chicken and duck lymphocytes using in vitro proliferation assays [5]. Chicken and turkey type I IFN have also been shown to be cross-reactive [6]. However, at least one report indicates that natural DuIFN has little or no cross-reactivity on chicken cells [7].

Immediately following infection of chickens with avian influenza virus (AIV) most cells begin to express proinflammatory cytokines, including IL-1β and IL-6, and Type I IFN genes, which results in a general antiviral response through the activation of a broad range of effector molecules, including Myxovirus resistance gene I (Mx), RNA-activated protein kinase (PKR) and 2',5'-oligoadenylate synthetases (OAS) [8-10]. Chickens have a single *Mx *gene (*Mx1*) that is induced by type I IFN [11]. The original evaluation of chicken Mx1 indicated the encoded protein lacked antiviral activity [12]. Ko et al., however, reported that the chicken *Mx1 *gene is highly polymorphic, and cDNAs of some but not all *Mx1 *alleles transfected into mouse 3T3 cells conferred protection against vesicular stomatitis virus (VSV) and highly pathogenic AI *in vitro *[13]. Recently, we demonstrated *in vivo *differences against AI in chickens with Mx1 variant alleles [14]. At least one report indicates duck Mx does not enhance resistance to influenza virus [15].

Beginning in April 2009, cases of acute respiratory disease were reported in humans and swine in Mexico caused by a novel H1N1 influenza A virus which was subsequently declared a pandemic [16]. Reports of the pH1N1 virus in turkeys was first observed in Chile, and later in North America on turkey breeder farms in Virginia and California, as well as Canada http://www.ars.usda.gov/2009h1n1/. The pH1N1 has also been detected in other species including dogs [17] and ferrets [18]. The pH1N1 is a triple reassortant virus containing genes from human (PB1), avian (PB2, PA), and swine (HA, NP, NA, M, NS) influenza viruses. The presence of avian and swine influenza virus genes in the pH1N1 raises the potential for infection in poultry following exposure to infected humans or swine. This is especially true for turkeys because of their known susceptibility to type A influenza viruses and the history of infection with triple reassortant viruses [19-22].

Our understanding of the immunological response to avian influenza by different avian species is largely unknown. In this study, we compared the growth kinetics of two avian influenza viruses containing both mammalian and avian origin genes (H1N1), or avian genes only (H5N9), in primary lung cell cultures from three common domestic poultry species (chicken, duck and turkey). The influence of chicken IFN-α on viral replication and host innate immune response genes following infection was also determined. Overall, chicken IFN-α reduced virus replication in all cell lines tested and decreased interferon and proinflammatory responses following AIV infection.

## Results

### Pretreatment with rChIFN-α inhibits AIV replication

**Figure 1:**
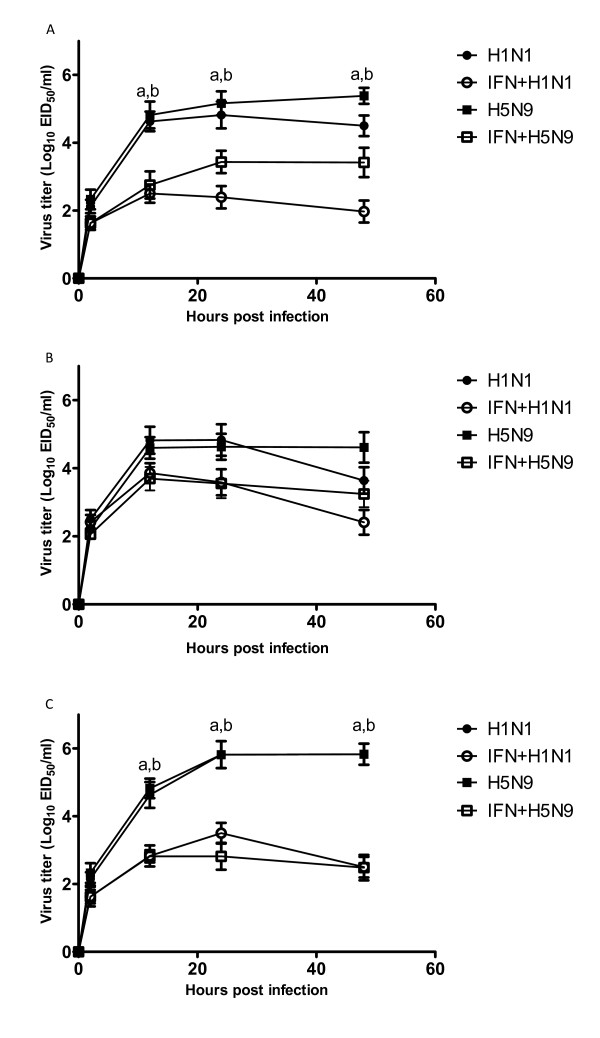
**Recombinant ChIFN-α reduces avian influenza virus replication**. Inhibition of avian influenza virus (H1N1 and H5N9) replication in primary lung cell cultures derived from chicken (A), duck (B), and turkey (C) after rChIFN-α (1000 U/ml) pretreatment in vitro. Cells were infected with A/turkey/Virginia/2009 H1N1 or A/turkey/Wisconsin/68 H5N9 at MOI 0.1. Supernatants were harvested at the times indicated and viral titers were determined following injection into SPF embryos. The mean (and standard deviations) of three independent experiments are shown. Different lowercase letters denote significance in titer following rChIFN-α treatment groups (within columns) (P < 0.05) as determined by one-way ANOVA. Statistical differences (P < 0.05) following treatment between virus groups are shown by lowercase letter.

To investigate the antiviral potential of chicken IFN-α against AIV *in vitro*, chicken, duck, and turkey primary lung cells were pretreated with 1000 U/ml rChIFN-α for18 hours prior to infection and viral growth was measured over 48 hours. As show in Figure (1A and 1C), at 2 hpi, reduced viral titers were first observed in chicken and turkey lung cell cultures pretreated with rChIFN-α. From 12 to 48 hpi, rChIFN-α significantly reduced virus replication compared to sham-treated cells (P < 0.05). At 24 and 48 hpi, virus growth was reduced by approximately 200-fold in both chicken and turkey lung cells. In duck lung cells, results demonstrate that pretreatment with rChIFN-α before AIV infection reduced virus replication, albeit to a lesser degree than observed with chicken or turkey cells (Figure 1B). At 2 hpi, no reduction in virus titer was observed. From 12 to 48 hpi, a reduction of virus titer was observed by approximately 30-fold in duck cells. Although no statistical difference was observed, a biological difference is apparent. These data demonstrate that rChIFN-α can reduce virus replication and is biologically active in other avian species.

### Pretreatment with rChIFN-α inhibits H1N1 and H5N9 virus NP expression

**Figure 2:**
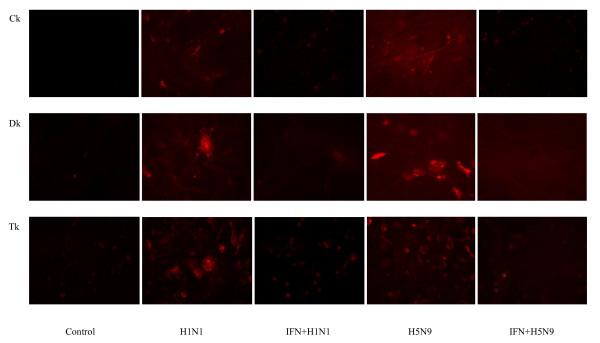
**Recombinant ChIFN-α inhibits pH1N1 and H5N9 virus nuclear protein expression**. Primary chicken, turkey, and duck lung cells were pretreated with or without rChIFN-α (1000 U/ml) for 18 h. Monolayers were infected with either H1N1 or H5N9 avian influenza virus (MOI = 0.1) for 1 h, and replaced with fresh media. After 24 hours, cells were fixed and viral antigens were reacted with mouse-derived monoclonal antibody (P13C11) specific for type A influenza virus nucleoprotein followed by detection with Texas Red-labeled goat anti-mouse IgG antibody. Magnification 400×.

To further demonstrate rChIFN-α pretreatment inhibits the replication of AIV, immunofluorescence assays to detect viral nuclear protein were performed. Figure 2 demonstrates decreased levels of viral NP expression at 24 hpi in the rChIFN-α treated chicken lung cells than untreated-infected cells with both H1N1 and H5N9 AIV. Similar staining patterns were observed for both duck and turkey lung cell cultures (data not shown). No staining was observed in any uninfected control cells. These results indicated that the pretreatment of cells with rChIFN-α strongly inhibits viral NP production.

### Reduced CPE following rChIFN-α pretreatment following AIV infection

**Figure 3:**
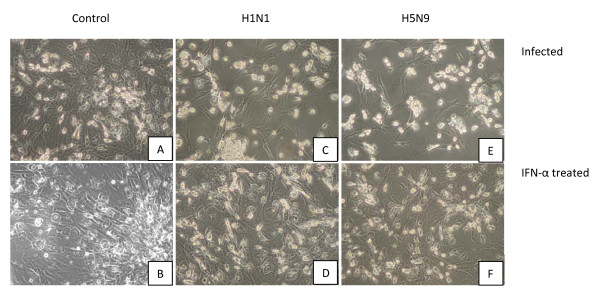
**Reduced cytopathic effect following rChIFN-α pretreatment following AIV infection**. Primary chicken lung cell monolayers were pretreated with 1000 U/ml of rChIFN-α and infected with either H1N1 of H5N9 at 0.1 MOI. Negative control cells include no treatment/no virus (A), and IFN-α only (B). Protection from cytopathic effect was observed in cells infected with virus only, H1N1 (C) and H5N9 (E), compared with IFN-α treated cells that were then infected with H1N1 (D) or H5N9 (F). At 24 hpi the monolayers were digitally photographed using an inverted microscope at 200× magnification (Olympus America Inc., Melville, NY).

The protective effect of rChIFN-α against CPE was determined in pretreated and virus-infected lung cell cultures. In uninfected-control chicken lung cells with or without IFN-α treatment, epithelial-like cell cultures were observed with clearly defined nucleus and cytoplasm in individual cells (Figure 3A and 3B). Morphologically, no CPE was observed for lung cells pretreated with rChIFN-α alone (Figure 3B). Additionally, chicken IFN-α was noncytotoxic based on cell viability after 48 hours exposure on all species tested (data not shown). Strong CPE was observed in both the H1N1 (Figure 3C) and H5N9 (Figure 3E) infected cells at 24 hpi, including decreased cell numbers and holes in monolayer with decreased direct cell-to-cell contact. However, pretreatment of monolayers with rChIFN-α abrogated the CPE observed in the virus infected cultures (Figure 3D and 3F). These results demonstrate that pretreatment of cells with rChIFN-α protected cells against virus induced CPE.

### Interferon-treatment attenuate the cytokine gene expression

**Figure 4:**
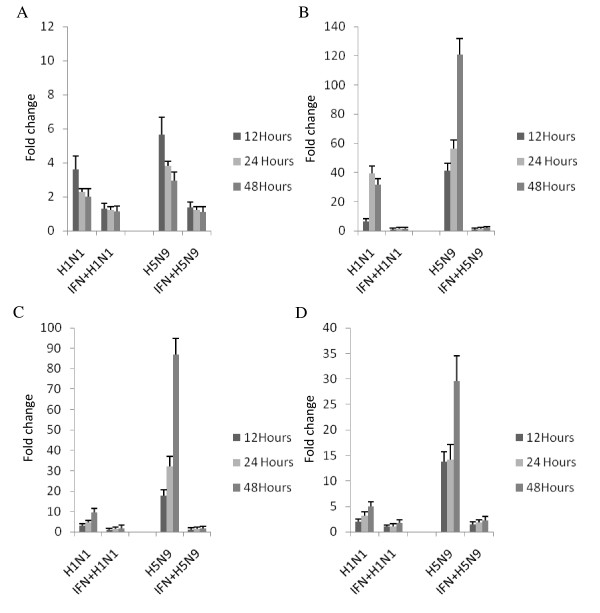
**Relative expression of select immune response genes following pretreatment of primary chicken lung cells with 1000 U/ml rChIFN-α, and infection with H1N1 or H5N9, compared to control (untreated/uninfected) cells**. The relative expression of IFN-α (A), Mx (B), IL-6 (C), and IL-1β (D) was measured following mock treatment at various time points post infection in three independent experiments. RNA from lung cells was normalized using the 28S house-keeping gene. Data are expressed as fold change in mRNA levels between interferon treated and infected cells compared with those from untreated and uninfected (negative control) cells.

We next investigated the effects of rChIFN-α on the innate immune response of avian lung cells to AIV using quantitative real-time RT-PCR. AIV infected and rChIFN-α pretreated cells were compared for induction of IFN-α, Mx, IL-1β and IL-6 mRNA at 12, 24 and 48 hpi. In all cell types tested, IFN-α pretreatment did not increase expression of the pro-inflammatory cytokines or IFN-α, but did up regulate Mx gene expression 2-5 fold (data not shown). In chicken lung cells, both H1N1 and H5N9 viruses induced an increased IFN-α response compared to sham-infected cells after infection that peaked early and declined over time (Figure 4). In contrast, rChIFN-α pretreatment resulted in a significant decrease of IFN-α expression after viral infection. Expression of the Mx gene was markedly higher in chicken lung cells after viral infection, especially in the H5N9 group which increased expression approximately 120-fold over the sham-infected cells. However, rChIFN-α pretreatment significantly reduced expression at all time points taken. Both viruses tested up regulated the proinflammatory cytokine genes, IL-1β and IL-6, after infection. Pretreatment with rChIFN-α significantly reduced expression compared to virus-infected cells. In general the H5N9 virus stimulated a higher innate immune response in chicken cells with the four genes examined than the H1N1 virus.

**Figure 5:**
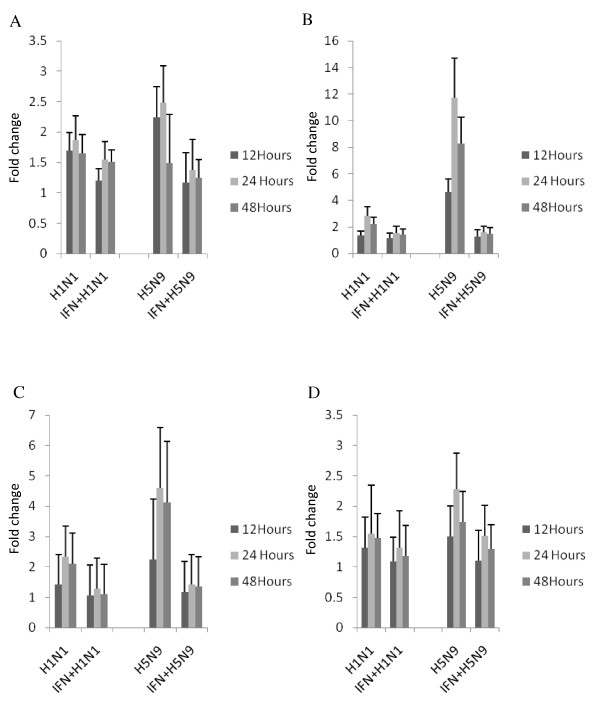
**Relative expression of select immune response genes following pretreatment of primary duck lung cells with 1000 U/ml rChIFN-α, and infection with H1N1 or H5N9, compared to control (untreated/uninfected) cells**. The relative expression of IFN-α (A), Mx (B), IL-6 (C), and IL-1β (D) was measured following mock treatment at various time points post infection in three independent experiments. RNA from lung cells was normalized using the GADPH house-keeping gene. Data are expressed as fold change in mRNA levels between interferon treated and infected cells compared with those from untreated and uninfected (negative control) cells.

In duck lung cells, neither H1N1 nor H5N9 viruses induced an increased IFN-α response compared to sham-infected cells (Figure 5). However, an increase in Mx expression was observed after H5N9, but not H1N1, infection that peaked with a 12-fold increase at 48 hpi. Pretreatment of cells with rChIFN-α significantly reduced Mx expression at all times tested. IL-6 gene expression was only up regulated following H5N9 infection, whereas the H1N1 virus did not induce up regulation of either IL-6 or IL-1β.

**Figure 6:**
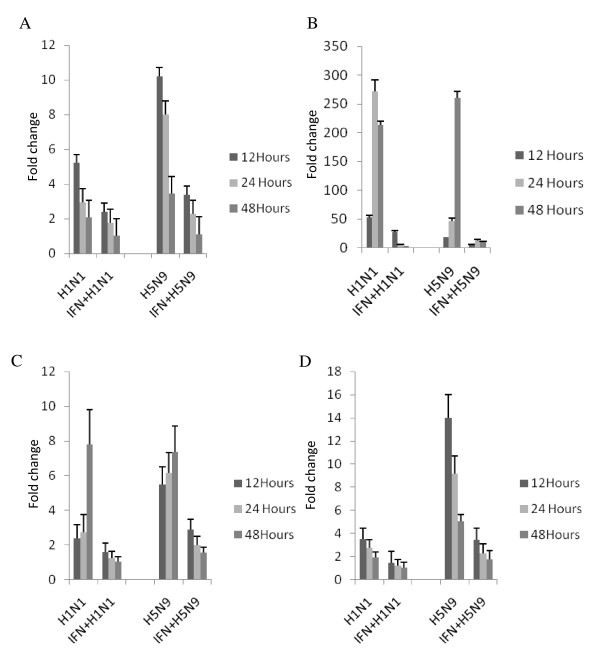
**Relative expression of select immune response genes following pretreatment of primary turkey lung cells with 1000 U/ml rChIFN-α, and infection with H1N1 or H5N9, compared to control (untreated/uninfected) cells**. The relative expression of IFN-α (A), Mx (B), IL-6 (C), and IL-1β (D) was measured following mock treatment at various time points post infection in three independent experiments. RNA from lung cells was normalized using the 28S house-keeping gene. Data are expressed as fold change in mRNA levels between interferon treated and infected cells compared with those from untreated and uninfected (negative control) cells.

In turkey lung cells, both H1N1 and H5N9 viruses induced an increased IFN-α response compared to sham-infected cells that also peaked early after infection and declined over time (Figure 6). rChIFN-α pretreatment significantly decreased the magnitude of IFN-α expression following H1N1 and H5N9 infection. Following virus infection, expression of the Mx gene was markedly high with both viruses inducing approximately 270-fold increase. Interestingly, pretreatment with rChIFN-α reduced Mx expression after virus infection, but not to the levels observed in either the chicken or duck cells, which were reduced to < 2 fold increase. Both viruses up regulated the IL-1β and IL-6, after infection in turkey cells, although the H5N9 stimulated a more robust response. Pretreatment with rChIFN-α significantly reduced the proinflammatory responses compared to virus-infected cells.

## Discussion

Avian influenza viruses present a permanent concern to the poultry industry and the recent emergence of pandemic H1N1 and highly pathogenic avian influenza H5/H7 subtypes serves as a reminder that influenza remains a severe threat throughout the world. Beside vaccination, there is an urgent need for new antiviral strategies to protect and treat against influenza. A significant portion of that strategy is to determine the influence of host-derived immune proteins on virus replication. Because AIV initially replicates on mucosal surfaces of avian species, including the respiratory tract, we chose to compare the immunological effect on replication in cells from this tissue. We report here that pretreatment with rChIFN-α before AIV infection reduced virus replication in chicken, duck and turkey lung cells.

Our study demonstrates that rChIFN-α reduces virus infection by limiting AIV replication, determined by decreased viral titers and decreased production of viral NP. The NP is important for maintaining the structure of the ribonucleoprotin complex, as well as genome replication by interacting with viral RNA [23-25]. Thus a reduction of viral protein synthesis appears to be at least on mechanism of anti-viral effect following rCHIFN-α treatment. Previously, three mechanisms of antiviral effects induced by IFN-α have been described in mice and humans, including activation of PKR, OAS, and Mx. Both PKR and OAS are important effector molecules that mediate a cellular response to foreign RNA structures [26,27]. Although neither PKR nor OAS induction was measure in this study, we show here that rChIFN-α pretreatment does up regulate Mx in chicken, turkey and duck cells, and positively correlated with decreasing virus replication. Further studies to determine the nature of viral inhibition with Mx proteins derived from different avian species are ongoing.

Previous studies have shown that chicken IFN-α administered to chicken by oral ingestion or intravenous injection can inhibit avian viruses including H9N2 AIV, Newcastle disease virus, infectious bursal disease virus, infectious bronchitis virus, Rous sarcoma virus, and Marek's disease virus [28,29]. In our studies, the presence of rChIFN-α significant limited the ability of these viruses to replicate, especially in the chicken and turkey lung cell cultures. Previous research indicates that chicken and turkey type I IFNs have been shown to be cross-reactive, such that some level of cross protection was not unexpected in the turkey lung cells. The rChIFN-α did not reduce titers on duck cells to the level observed in the chicken or turkey lung cells. However, a moderate biological effect (> 1 log_10 _reduction) was evident in the absence of statistical differences. Because of the amino acid differences between chicken and duck IFN, it seems likely that rChIFN-α is not as efficient at inducing an antiviral effect in this species. Whether this effect is due to decreased IFN-α receptor affinity or downstream transcription factor activation for cytokine expression remains to be determined.

When virus replication was compared between the three kinds of primary lung cells, we observed that both viruses replicate to the highest titers on the turkey lung cells, followed by chicken lung cells and duck lung cells. This data suggest that turkey may be more susceptible to H1N1 and H5N9 virus than white leghorn chickens and Pekin ducks. This result may not be unexpected since both viruses are of turkey origin and maybe be better adapted for this species. These results also highlight the role of turkeys as intermediate host in the transmission of influenza viruses from domestic poultry to humans. The detection of α2,3 (avian type) and α2,6 (mammalian type) sialic-acid-linked receptors in the turkeys further indicate that this species can replicate both avian and mammalian viruses [19,30,31]. This is consistent with some reports that turkeys were more susceptible to disease from LPAI virus than chickens and ducks [32-34].

Interestingly, interferon treatment significantly decreased the interferon and proinflammatory response after viral infection. The decreased proinflammatory response positively correlated with decreased virus replication, and may explain the reason for this observation. In addition, infection with the H1N1 virus produced a decreased expression of the innate immune genes tested, including Mx, IL-1β and IL-6 than observed with the H5N9 virus. This result is consistent with some recent reports that indicate pandemic H1N1 isolates induce weaker cytokines responses in human cells [35,36]. In general, a robust cytokine response is associated with highly pathogenic influenza viruses, including H5N1 viruses, and it is thought that this cytokine dysregulation may contribute to disease severity [37]. Our results with low pathogenic AI suggests that a suboptimal cytokine response maybe in part explain how H1N1 could escape the innate immune defense by impeding cytokine response. This phenomenon maybe characteristic of low pathogenic AI viruses as well since they also have demonstrated the ability to limit the host's antiviral Mx response in chickens in vivo [38]. Data presented here will contribute to a better understanding of the avian host response to the low pathogenic AI viruses, and our model of testing primary avian lung cell cultures will be useful for monitoring new AIV isolates for changes in innate immune modulation.

## Conclusions

The present study demonstrates that pretreatment with rChIFN-α prior to infection with the pandemic H1N1 and H5N9 avian influenza viruses not only significantly reduced virus replication in both chicken-and turkey-origin lung cells, and to a lesser degree the duck-origin lung cells, but also significantly decreased the interferon and proinflammatory response after viral infection. Thus, under the scenario of avian influenza, rChIFN-α might provide an additional option in the prevention and therapy against low pathogenic AIV infection. Similar conclusions were recently described following oral administration of rChIFN-α and H9N2 AIV infection [28]. Further investigation into the molecular mechanisms of protection induced by chicken IFN-α are underway and will add more information on its anti-viral role.

## Methods

### Virus and cell culture infection

The low pathogenic AI viruses H1N1 A/turkey/Virginia/SEP-4/2009 (H1N1) and H5N9 A/turkey/Wisconsin/68 (H5N9) were propagated in the allantoic cavities of 11 day of embryonating specific pathogen free (SPF) turkey eggs. Viral titers were determined as previously described [39]. All experiments using infectious virus were conducted in a biosafety level 2 (BSL-2) facilities at the Southeast Poultry Research Laboratory (SEPRL), Agricultural Research Service, United States Department of Agriculture (USDA) in Athens, Georgia.

### Cells isolation and culture

Avian lung primary cells were isolated as described previously with minor modifications [40]. Briefly, lungs from four-week-old specific pathogen-free (SPF) white leghorn chickens, six-week-old SPF Beltsville White turkeys and eight-week-old commercial Pekin ducks were aseptically collected and trypsinized before culturing in 12-well tissue culture plate coated with 0.01% (w/v) calf skin collagen (Sigma Chemical Co., St. Louis, Mo.). Cells were cultured at 1×10^6 ^lung cells per ml of Dulbecco's modified Eagle's medium (DMEM) supplemented with 1% L-glutamine, 1% sodium pyruvate, 1% MEM nonessential amino acids, 1% antibiotic-antimycotic solution (Sigma), and 10% chicken serum in a humidified incubator at 37°C. All animals used in these studies were housed and handled in compliance with our Institutional Animal Care and Use Committee guidelines and procedures.

### rChIFN-α treatment and virus infection

Lung cells were grown overnight in 12-well plates (Fisher Scientific, Atlanta, Ga). Immediately before IFN treatment, the cells were washed with warm PBS and subsequently treated with 1000 U/ml of recombinant chicken IFN-α (rChIFN-α, AbD Serotec Co., Oxford, UK) for 18 hours in MEM containing 0.2% bovine albumin (BA) and antibiotics. After treatment, rChIFN-α was aspirated and cells were washed with PBS. Thereafter, cells were inoculated with H1N1 or H5N9 at a multiplicity of infection (MOI) of 0.1 diluted in DMEM containing antibiotics for one hour at 37°C with gentle agitation every 10 minutes. After one hour of incubation, unabsorbed virus was removed and cells were washed with PBS. Fresh media supplemented with 0.01 μg/ml TPCK trypsin (Sigma) were added per well and the plate were incubated at 37°C and 5% CO2. At 0, 2, 12, 24 and 48 hours post infection (hpi), supernatants were collected and stored at -80°C until used for titrations. Lung cells were harvested for RNA extraction at 12, 24, 48 hpi. Virus titers was determined using the method of Reed and Muench and expressed as log10 50% embryo infectious dose (EID_50_) [41]. Controls included one plate without virus and another one plate without either rChIFN-α or virus. The plate was then incubated under the same conditions as above.

### Immunofluorescence assays for virus nuclear protein (NP)

To analyze antiviral effect of rChIFN-α on virus replication, primary avian lung cells were cultured on glass cover slips in 24-well plate. After rChIFN-α treatment and virus infection for 24 hours (as described above), cells were washed with PBS twice, fixed and permeabilized with ice-cold methanol. Viral antigens were detected with mouse-derived monoclonal antibody specific for a type A influenza virus nucleoprotein (developed at Southeast Poultry Research Laboratory, USDA) [42]. Cells were then stained with TRITC-conjugated anti-mouse IgG antibody (Sigma). The stained cells were visualized with immunofluorescence microscopy (Olympus America Inc., Melville, NY) under 400× magnification.

### Cytopathic effect (CPE) of rChIFN-α pretreatment on virus infection

To visually compare virus inhibition following rChIFN-α treatment, primary avian lung cells were seeded as above on glass cover slips in 24-well plate. Following rChIFN-α treatment, cells were virally infected as described above. After 24 hours, the cells were fixed with ice-cold acetone and CPE was visualized by inverted microscopy (Olympus).

### Isolation of RNA and analysis of cytokine expression by real-time RT-PCR (RRT-PCR)

RNA was extracted using the RNeasy mini kit (Qiagen) in accordance with the manufacturer's instructions. Relative cytokine expression in lung cells was examined by RRT-PCR. IL-1β, IL-6, IFN-α, and Mx expression were determined as previously described [14,43]. Briefly, quantitative RRT-PCR was performed for each sample in triplicate in a total volume of 25 μl, consisting of 12.5 μl iQ Sybrgreen supermix (Bio-Rad Laboratories, Los Angeles, CA, USA) with 1 μl of each primer at concentration of 10 pmol/μl, 5.5 μl RNase/DNase-free water, and 5 μl diluted RNA. PCR conditions were the same for each targeted gene and are as follows: 10 min at 50°C, 95°C for 5 min, followed by 45 cycles of 95°C for 10 s and 56°C for 30 s. Primers for chicken 28 s, IFN-α, IL-1β [14]; turkey 28 s, IL-1β, IL-6 [44]; duck GAPDH, IL-1β, IL-6, IFN-α [45] have been previously described. The other primers were designed using the Primer Express software program (Applied Biosystems, Foster City, California, USA) and sequences used in this study for individual avian species are presented in Table 1. The specificity for each primer set was tested by both subjecting the PCR products to 1.5% agarose gel electrophoresis (data not shown) and analyzing the melting curve in the iCycler iQ real-time PCR detection system (Bio-Rad) after each real-time PCR reaction.

**Table 1:** Real-time quantitative RT-PCR primers used in this study^1^

RNA target	Primer Sequence (5'-3')	Species
28S	F: GGCGAAGCCAGAGGAAACT	C, T
	R: GACGACCGATTTGCACGTC	
GADPH	F: ATGTTCGTGATGGGTGTGAA	D
	R: CTGTCTTCGTGTGTGGCTGT	
IFN-α	F: GACAGCCAACGCCAAAGC	C, T
	R: GTCGCTGCTGTCCAAGCATT	
IFN-α	F: TCCTCCAACACCTCTTCGAC	D
	R: GGGCTGTAGGTGTGGTTCTG	
Mx	F: CAGGACATCAACGACAATCT	C, T
	R: TTGCCAGATGAGGGATAGTA	
Mx	F: TCAATCACTTTCCTCACCAG	D
	R: CCTTCTGCTTGTGTTGAGAC	
IL-6	F: GCGAGAACAGCATGGAGATG	C
	R: GTAGGTCTGAAAGGCGAACAG	
IL-6	F: GCTCGCCGGCTTCGA	T
	R: GGTAGGTCTGAAAGGCGAACAG	
IL-6	F: TTCGACGAGGAGAAATGCTT	D
	R: CCTTATCGTCGTTGCCAGAT	
IL-1β	F: ACATGTCGTGTGTGATGAG	C
	R: CAG CCGGTAGAAGATGAAGC	
IL-1β	F: GCTCTACATGTCGTGTGTGATGAG	T
	R: TGTCGATGTCCCGCATGA	
IL-1β	F: TCGACATCAACCAGAAGTGC	D
	R: GAGCTTGTAGCCCTTGATGC	

^**1 **^F, forward primer; R, reverse primer; C, chicken; T, turkey; D, duck.

RNA from individual lung cell sample was normalized using the 28S for chicken and turkey and GAPDH for duck. For each gene, amplification was verified using four 10-fold serial dilutions of standard spleen cell RNA in the same PCR run. Expression was determined by the standard curve method [46]. Data are expressed as fold change in cytokine messenger RNA (mRNA) levels in infected groups compared with those from uninfected, untreated groups.

### Statistical analyses

Data are expressed as the mean ± standard error. Statistical differences were analyzed with Tukey one-way ANOVA using Prism 5 (GraphPad Co., San Diego, CA). All statistical tests were performed using P ≤ 0.05.

## Competing interests

The authors declare that they have no competing interests.

## Authors' contributions

HJ and DRK carried out virus growth on cell culture as well as RRT-PCR for avian cytokines. HJ performed immunohistochemistry and cytology of primary avian lung cultures. HY participated in study design and coordination. HJ and DRK wrote the manuscript. All authors approved the final manuscript.
